# Biogenic Selenium Nanoparticles Functionalized with Natural Polymers or Phytochemicals for Targeted Disruption of *Candida* spp. Biofilms on Denture Materials: A Systematic Review

**DOI:** 10.3390/jfb17050216

**Published:** 2026-05-01

**Authors:** Zofia Stefanik, Paweł Ścierski, Maciej Dobrzyński, Natalia Stefanik, Magdalena Antonowicz-Hüpsch, Rafał Wiench

**Affiliations:** 1 Department of Periodontal Diseases and Oral Mucosa Diseases, Faculty of Medical Sciences in Zabrze, Medical University of Silesia, 40-055 Katowice, Poland; nstefanik@sum.edu.pl; 2 Department of Otorhinolaryngology and Laryngological Oncology in Zabrze, Medical University of Silesia, 41-800 Zabrze, Poland; s87914@365.sum.edu.pl; 3 Department of Pediatric Dentistry and Preclinical Dentistry, Faculty of Medicine and Dentistry, Wrocław Medical University, 50-425 Wroclaw, Poland; maciej.dobrzynski@umw.edu.pl; 4 Department of Biomaterials and Medical Devices Engineering, Faculty of Biomedical Engineering, Silesian University of Technology, 41-800 Zabrze, Poland; magdalena.antonowicz@polsl.pl

**Keywords:** biogenic selenium nanoparticles, *Candida albicans*, denture stomatitis, biofilms, polymethyl methacrylate, natural polymers, phytochemicals, antifungal nanomaterials

## Abstract

Background: Denture stomatitis is strongly associated with *Candida* biofilms on prosthetic surfaces and remains difficult to manage due to biofilm persistence and antifungal resistance. Selenium-based nanomaterials, particularly biogenic selenium nanoparticles (SeNPs) functionalized with natural polymers or phytochemicals, have emerged as potential material-centered strategies for biofilm control. Objective: To systematically evaluate the antifungal and antibiofilm effects of selenium-based nanomaterials on *Candida* biofilms in the context of denture materials. Methods: A systematic review was conducted in accordance with the PRISMA guidelines and registered in PROSPERO. Multiple databases were searched from inception without language restrictions. Eligible studies included experimental investigations of biogenic or functionalized SeNPs or organoselenium compounds targeting *Candida* biofilms on denture materials or in relevant in vitro models. A qualitative synthesis was performed due to anticipated heterogeneity. Results: Eleven studies met the inclusion criteria. Of these, four studies directly evaluated selenium-based interventions on denture materials, while seven provided supporting mechanistic evidence using in vitro models on non-denture substrates. Across denture-related studies, selenium-based modifications reduced fungal adhesion, biofilm biomass, and colony-forming units, without detrimental effects on material properties. Functionalization with polymers or phytochemicals was associated with enhanced antifungal activity and nanoparticle stability. Mechanistic studies suggested multimodal antifungal effects, including membrane disruption, inhibition of virulence factors, and modulation of biofilm-related pathways. Methodological quality was moderate, with common limitations in reporting and experimental standardization. Conclusions: Functionalized biogenic SeNPs show promising antifungal and antibiofilm activity against *Candida* in preclinical denture-related models. However, all available evidence is in vitro, with no in vivo or clinical studies identified. Substantial heterogeneity and limited long-term safety data preclude clinical recommendations. Further research should focus on standardized methodologies, clinically relevant in vivo models, and controlled clinical trials to assess translational potential.

## 1. Introduction

### 1.1. Rationale

Denture stomatitis is a common inflammatory condition affecting individuals wearing removable prostheses, with reported prevalence ranging from 17% to 75% across populations [1,2]. Clinically, it manifests as erythema and inflammation of the mucosa in direct contact with the denture base and is frequently associated with discomfort, impaired prosthesis tolerance, and reduced quality of life, particularly in elderly and medically compromised patients [1,3].

The condition is primarily driven by microbial dysbiosis, with a central role attributed to *Candida albicans* biofilm formation on denture materials, especially polymethyl methacrylate (PMMA) [1,3,4,5]. Importantly, denture stomatitis reflects a broader disruption of the oral microbiome rather than an isolated pathology. This dysbiotic biofilm environment contributes to the development or coexistence of other oral conditions, including angular cheilitis, dental caries, and periodontitis, which share common microbial and host-related risk factors such as poor oral hygiene, reduced salivary flow, and immunocompromised status [3,4]. In vulnerable populations, particularly frail older adults, this microbial burden has also been associated with an increased risk of aspiration pneumonia through oral–respiratory translocation of pathogens [4].

*Candida* biofilms exhibit markedly increased tolerance to antifungal agents compared with planktonic cells. This resistance is mediated by multiple mechanisms, including extracellular matrix-associated drug sequestration, altered metabolic activity, and phenotypic adaptation, resulting in substantially reduced susceptibility to commonly used antifungal agents such as amphotericin B, nystatin, fluconazole, and chlorhexidine [6,7,8,9,10]. These properties contribute to persistence and recurrence, limiting the long-term effectiveness of conventional antifungal therapies [3,11,12]. Given these limitations, increasing attention has been directed toward material-centered strategies that target biofilm formation directly at the denture surface. Incorporation of antimicrobial agents into denture base materials or the application of bioactive coatings offers the potential for sustained, localized activity that is less dependent on patient adherence [1]. However, previously investigated nanomaterials, particularly metallic and metal oxide nanoparticles, raise concerns regarding cytotoxicity, long-term stability, environmental impact, and potential induction of resistance.

Biogenic selenium nanoparticles (SeNPs) have emerged as a potential alternative within this context. These nanoparticles, synthesized via biological or green methods, have demonstrated antifungal and antibiofilm activity together with a relatively favorable biocompatibility profile compared with conventionally synthesized nanomaterials [13,14,15]. Their antimicrobial activity is considered to involve multiple pathways, including disruption of fungal cell structures, induction of oxidative stress, and interference with biofilm development and virulence. Functionalization with natural polymers (e.g., chitosan) or phytochemicals (e.g., plant extracts) may further enhance nanoparticle stability, surface interaction, and biological activity [14,16,17,18]. Despite these promising properties, the engineering challenges associated with functionalizing selenium nanoparticles for effective and stable integration into denture materials remain insufficiently addressed. These include achieving reproducible synthesis, ensuring stable incorporation or surface binding, maintaining mechanical and physicochemical properties of the denture substrate, and controlling nanoparticle release and long-term biocompatibility. Furthermore, the available evidence is highly heterogeneous, spanning material science, microbiology, and preclinical biofilm models, which complicates direct translation into clinical prosthodontic applications [19,20,21,22,23].

### 1.2. Objectives

The aim of this systematic review is to critically appraise and synthesize the available evidence on biogenic selenium nanoparticles (SeNPs), particularly those functionalized with natural polymers or phytochemicals, for the control of *Candida* spp. biofilms on denture materials.

This review focuses specifically on:

(i) synthesis approaches and physicochemical characteristics of functionalized SeNPs;
(ii) antifungal and antibiofilm efficacy on denture-related substrates;
(iii) effects on denture material properties and stability; and
(iv) cytotoxicity and biocompatibility profiles in relevant experimental models.

This systematic review applies predefined eligibility criteria, structured data extraction, and a domain-based risk-of-bias assessment to provide a critical and transparent evaluation of the evidence. Mechanistic studies not conducted on denture materials are included only to support biological plausibility and are not considered direct evidence for clinical application.

The objective is therefore to identify methodological limitations, sources of heterogeneity, and key translational gaps that must be addressed before functionalized selenium nanoparticles can be considered for clinical use in denture-associated *Candida* infections.

## 2. Methods

This systematic review was conducted and reported in accordance with the Preferred Reporting Items for Systematic Reviews and Meta-Analyses (PRISMA) 2020 statement [24]. The review protocol was prospectively registered in the PROSPERO database (CRD420261321297) [25,26]. The PRISMA Checklist can be found in File S1.

### 2.1. Focused Question

The review question was structured using the PICO framework:

- Population (P): In vitro, ex vivo, or in vivo models of *Candida* spp. biofilms formed on denture or dental prosthetic materials
- Intervention (I): Biogenic (green-synthesized) selenium nanoparticles (SeNPs) functionalized with natural polymers or phytochemicals
- Comparison (C): Untreated controls, conventional antifungal agents, or non-functionalized/chemically synthesized selenium nanoparticles
- Outcome (O): Antifungal or antibiofilm efficacy, material properties, nanoparticle stability, and cytotoxicity/biocompatibility

### 2.2. Search Strategy

A comprehensive search was performed in four electronic databases—PubMed (MEDLINE), Embase, Scopus, and the Cochrane Library—from inception to 19 January 2026. The search strategy combined controlled vocabulary (MeSH) and free-text terms related to selenium nanoparticles, *Candida* spp., biofilms, and denture materials. No restrictions on language or publication year were applied at the search stage. Non-English articles were screened and, where necessary, translated using a combination of automated tools and institutional resources. Reference lists of included studies and relevant reviews were screened manually to identify additional records. The full search syntax for each database is provided in Table 1.

### 2.3. Eligibility Criteria

Eligibility criteria were defined a priori and applied during both title/abstract and full-text screening. Only peer-reviewed primary studies presenting original experimental data (quantitative or qualitative), including in vitro, ex vivo, or in vivo investigations, were considered. Reviews, editorials, opinion papers, and conference abstracts without full datasets were excluded.

Inclusion criteria:

- Original peer-reviewed experimental studies (in vitro, ex vivo, or in vivo);
- Use of selenium nanoparticles produced via biogenic or green synthesis (e.g., plant-mediated, microbial, or natural polymer-assisted);
- Functionalization, stabilization, or incorporation of SeNPs with natural polymers (e.g., chitosan, alginate, cellulose) or phytochemicals (e.g., plant extracts, flavonoids);
- Evaluation of *Candida* spp. biofilms (formation, adhesion, growth, or disruption)
- Use of denture base materials or clinically relevant prosthetic substrates (e.g., PMMA, acrylic resins, 3D-printed resins), or mechanistic studies directly relevant to these systems;
- Reporting at least one relevant outcome (antifungal efficacy, biofilm reduction, material properties, stability, or cytotoxicity).

Exclusion criteria:

- Studies not involving *Candida* biofilms (e.g., planktonic-only models);
- Studies using exclusively chemically synthesized selenium nanoparticles without biogenic or natural functionalization;
- Studies not involving denture materials or lacking clear translational relevance to prosthetic substrates;
- Non-original publications (reviews, editorials, letters, conference abstracts without full data, theses);
- Studies focusing exclusively on bacterial biofilms or non-oral biofilm systems.

### 2.4. Study Selection and Data Management

All retrieved records were imported into EndNote 21 (Clarivate Analytics, Philadelphia, PA, USA) for reference management and deduplication. Automated duplicate removal was followed by manual verification.

Screening was conducted independently by two reviewers in two stages:

(i) title and abstract screening;
(ii) full-text assessment.

Discrepancies were resolved through discussion, and when necessary, by consultation with a third reviewer. Reasons for exclusion at the full-text stage were recorded systematically.

### 2.5. Data Extraction

Data extraction was performed independently by two reviewers using a standardized and pilot-tested extraction form. The form was refined after initial testing on a subset of included studies to ensure consistency and completeness.

Extracted data included:

- Study characteristics: author, year, country, study design.
- Experimental model: in vitro, ex vivo, or in vivo; biofilm model details.
- *Candida* species and strains (with explicit identification of *C. albicans* dominance where applicable).
- Denture material type and preparation.
- Nanoparticle characteristics: synthesis method, functionalization type, size, morphology, surface charge.
- Intervention details: concentration, exposure time, application method.
- Comparator groups.
- Outcomes: antibiofilm/antifungal effects, material properties, stability, cytotoxicity.

Given that the included evidence was dominated by *Candida albicans*, this was explicitly recorded during extraction. Non-*albicans* species, when present, were analyzed descriptively but not as a separate quantitative subgroup due to limited data [27,28,29,30,31,32,33,34,35,36,37,38].

### 2.6. Risk of Bias and Quality Assessment

Methodological quality was assessed using an adapted domain-based appraisal tool designed for in vitro nanobiomaterial research [39,40,41]. The tool included nine domains:

(1) synthesis characterization;
(2) physicochemical characterization;
(3) substrate standardization;
(4) biofilm model description;
(5) comparators;
(6) outcome measures;
(7) bias-control measures;
(8) statistical analysis;
(9) transparency/conflicts of interest.

Each domain was scored as 1 (adequately reported) or 0 (unclear/inadequate), yielding a maximum score of 9. Studies were categorized as having low (7–9), moderate (4–6), or high (0–3) risk of bias.

Disagreements between reviewers were infrequent and resolved through discussion; no persistent disagreements required arbitration beyond consensus.

### 2.7. Data Synthesis

Given the substantial heterogeneity across studies in terms of nanoparticle synthesis methods, functionalization strategies, denture materials, biofilm models, and outcome measures, a structured qualitative synthesis was undertaken. Studies were grouped according to:

- type of functionalization (natural polymer vs. phytochemical);
- denture material;
- *Candida* species and experimental model.

Although meta-analysis was planned when at least three studies reported comparable quantitative outcomes, no quantitative synthesis was ultimately feasible due to marked heterogeneity in:

- nanoparticle formulations (size, coating, concentration);
- biofilm models (early vs. mature, mono- vs. multi-species);
- outcome measures (CFU counts, biomass, gene expression, microscopy); and
- experimental conditions.

As a result, pooling of data would not have been methodologically appropriate or clinically interpretable.

Mechanistic studies not conducted on denture materials were included solely to support biological plausibility and were analyzed separately from denture-specific evidence. They were not considered direct evidence for clinical translation. The findings are therefore presented as a systematic, structured narrative synthesis with critical appraisal of methodological quality and translational relevance.

## 3. Results

### 3.1. Study Selection

**Figure 1:**
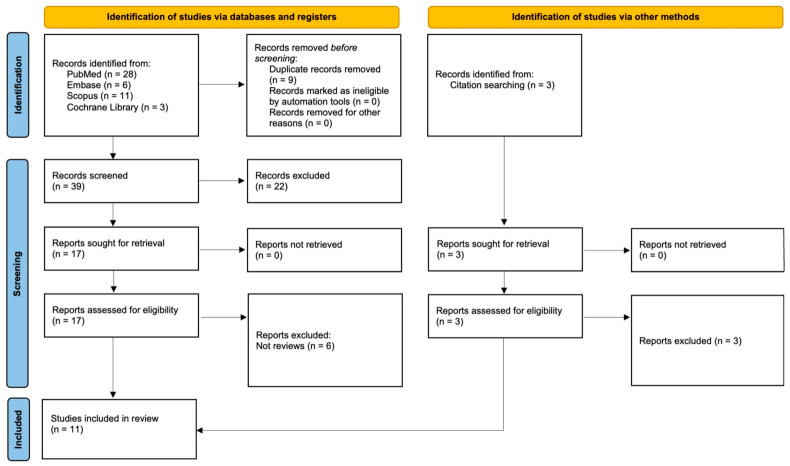
PRISMA 2020 flow diagram showing the study selection process.

The PRISMA 2020 flow diagram (Figure 1) summarizes the study selection process. A total of 48 records were identified through database searching (PubMed *n* = 28, Embase *n* = 6, Scopus *n* = 11, Cochrane Library *n* = 3), with an additional 3 records identified through citation searching. After removal of 9 duplicates, 39 records underwent title and abstract screening, of which 22 were excluded. Seventeen full-text articles were assessed for eligibility, and six were excluded (all review articles). All three records identified through citation searching were also excluded as reviews. Ultimately, 11 studies met the inclusion criteria and were included in the qualitative synthesis. The numbers reported in the text correspond exactly to those presented in Figure 1.

### 3.2. Classification of Included Studies

The included studies were categorized into two distinct groups based on their direct relevance to denture materials [42,43,44,45,46,47,48]:

- Denture material studies (n = 4): Experimental investigations evaluating selenium-based interventions directly on denture base materials (e.g., PMMA, 3D-printed resins) [19,23,45,48].
- Supporting mechanistic studies (n = 7): In vitro studies investigating functionalized selenium nanoparticles on non-denture substrates to explore antifungal mechanisms, virulence modulation, or nanoparticle properties [15,16,17,43,44,47,49,50,51].

This distinction was applied a priori and maintained throughout the analysis. Mechanistic studies were not considered direct evidence for denture applications and were used solely to support biological plausibility.

A structured summary of all included studies, with explicit labeling of study type, is provided in Table 2.

### 3.3. General Characteristics of Included Studies

All included studies were experimental, conducted in vitro. No in vivo denture models or randomized clinical trials were identified. Publication years ranged from 2018 to 2025, with increased frequency after 2020. The predominant fungal species investigated was *Candida albicans*, which was reported in all denture-related studies and the majority of mechanistic studies. Data on non-*albicans* species were limited and heterogeneous, precluding separate analysis. Biofilm models varied substantially across studies, including:

- early adhesion models (initial colonization);
- intermediate biofilm formation models; and
- mature biofilm systems.

Denture substrates included conventional heat-polymerized PMMA, acrylic resins, and additively manufactured (3D-printed) denture base materials [19,23]. No standardized protocol for specimen preparation or aging was consistently applied across studies. A summary of the included studies is show in Table 2.

### 3.4. Selenium-Based Strategies: Preventive vs. Reactive Approaches

Two principal application strategies were identified in denture-related studies:

- Preventive strategies: Surface coatings or material modifications designed to inhibit initial fungal adhesion and early biofilm formation (e.g., organoselenium coatings on acrylic surfaces) [45,48].
- Reactive strategies: Incorporation of selenium nanoparticles within denture materials aimed at reducing established biofilms or limiting biofilm maturation (e.g., SeNP-modified PMMA resins) [19,23].

Preventive approaches were primarily evaluated using early-stage biofilm models, whereas reactive strategies were more commonly assessed in established or mature biofilm systems. No study directly compared preventive and reactive strategies within the same experimental framework.

### 3.5. Selenium-Based Materials and Functionalization

Denture-related studies employed two main material strategies:

- Organoselenium surface coatings or sealants [45,48].
- SeNP incorporation into denture base resins [19,23].

Mechanistic studies predominantly used biogenic selenium nanoparticles, synthesized via plant-mediated or polymer-assisted approaches [15,16,17]. Functionalization strategies included natural polymers (e.g., chitosan) and phytochemicals (e.g., ginger extract, plant-derived compounds). Functionalization was consistently associated with improved nanoparticle dispersion and colloidal stability, although characterization methods and reporting were heterogeneous across studies.

### 3.6. Antifungal and Antibiofilm Outcomes

Across denture material studies, selenium-based interventions demonstrated:

- reduction in *Candida* biofilm biomass and colony-forming units;
- inhibition of fungal adhesion to denture surfaces; and
- dose-dependent antifungal effects in nanoparticle-modified resins [19,23,45,48].

Preventive coatings primarily reduced early adhesion, whereas nanoparticle-modified materials demonstrated effects across both early and more developed biofilms.

No study reported direct comparisons with standardized antifungal treatment protocols under clinically equivalent conditions. Outcome measures varied widely, including CFU counts, metabolic assays, and microscopy-based assessments, limiting cross-study comparability.

### 3.7. Mechanistic Patterns

Two mechanistic patterns were identified:

- Single-agent effects: Antifungal activity attributable to selenium nanoparticles or organoselenium compounds alone, typically involving biofilm inhibition or structural disruption.
- Synergistic effects: Enhanced antifungal activity observed when selenium nanoparticles were combined with functionalizing agents such as chitosan, phytochemicals, or antifungal drugs (e.g., nystatin) [14,15,16,17,47,51].

Synergistic effects were predominantly reported in mechanistic studies rather than denture material studies. Quantitative comparisons between single-agent and combined approaches were not consistently performed.

### 3.8. Effects on Denture Material Properties and Stability

In denture-related studies, selenium-based modifications did not demonstrate measurable adverse effects on:

- mechanical strength;
- surface integrity; or
- material morphology [19,23,45].

Where assessed, selenium compounds remained stably incorporated or bound to the material, with no detectable leaching under experimental conditions. However, testing protocols for aging, mechanical stress, and long-term exposure were limited and not standardized.

### 3.9. Cytotoxicity and Biocompatibility

Biocompatibility data were limited and derived primarily from mechanistic studies. Functionalized selenium nanoparticles generally demonstrated:

- low cytotoxicity toward mammalian cells; and
- acceptable short-term biocompatibility profiles.

However, there was substantial heterogeneity in cell models, exposure conditions, and outcome measures. No long-term or clinically relevant mucosal models were identified.

### 3.10. Risk of Bias and Methodological Quality

The methodological quality of included studies was assessed using the predefined 9-domain scoring system (maximum score = 9).

- Low risk of bias (7–9 points): 4 studies [41,42,45,48];
- Moderate risk of bias (4–6 points): 7 studies [43,44,46,47,49,50,51].

The most frequently underreported domains were:

- bias-control measures (randomization, blinding);
- detailed physicochemical characterization of nanoparticles; and
- sample size justification and statistical rigor.

Domains related to basic experimental design (e.g., biofilm model description, comparator inclusion) were more consistently reported. Overall, the included evidence demonstrates moderate methodological quality, with recurrent limitations affecting reproducibility and comparability across studies. The results of this is shown in Table 3.

## 4. Discussion

This systematic review evaluated selenium-based strategies for the control of *Candida* biofilms on denture materials, with a clear distinction between direct denture material evidence and supporting mechanistic studies. The available evidence indicates that functionalized selenium nanoparticles and organoselenium surface modifications demonstrate antifungal and antibiofilm activity in vitro; however, the strength of this conclusion is constrained by the limited number of denture-specific studies and the absence of in vivo or clinical data.

### 4.1. Preventive Versus Reactive Strategies

A central issue emerging from this analysis is whether selenium-based nanomaterials should be conceptualized primarily as preventive or reactive interventions. The included denture material studies suggest that both strategies are represented, but not systematically compared. Preventive approaches, based on surface coatings or material functionalization, aim to inhibit initial fungal adhesion and early biofilm formation [45,48]. These strategies are conceptually aligned with the pathogenesis of denture stomatitis, where biofilm establishment on prosthetic surfaces is a critical initiating step [1]. In contrast, reactive approaches, such as selenium nanoparticle incorporation into denture resins, target already established biofilms and aim to reduce biomass or metabolic activity [19,23]. The current evidence does not allow determination of superiority between these approaches. However, given that most denture-related biofilm models focus on early adhesion, the available data are more consistent with a preventive strategy. This distinction is clinically relevant, as preventive material-based strategies could theoretically reduce reliance on patient-dependent antifungal regimens, although this remains unproven in clinical settings.

### 4.2. Denture Materials and Biofilm Models

The antifungal efficacy of selenium-based interventions appears consistent across different denture substrates, including conventional PMMA and additively manufactured resins [19,23,24,25,26,27,28,29,30]. This suggests that the observed effects are not limited to a specific material class. However, methodological heterogeneity substantially limits comparability [31,32,33,34,35,36,37,38]

Biofilm models varied widely across studies, ranging from early adhesion assays to mature biofilm systems [39,40,41,42,43,44,45,46,47,48,49,50]. Importantly, most studies used mono-species *Candida albicans* models, which do not reflect the polymicrobial nature of denture biofilms in vivo [51,52,53,54,55,56,57,58,59,60,61,62,63,64,65,66,67,68,69,70,71,72,73,74,75,76]. The lack of standardized biofilm protocols, including differences in incubation time, nutrient conditions, and outcome measures, introduces significant variability and reduces external validity. Furthermore, denture materials were not consistently subjected to clinically relevant aging conditions such as mechanical loading, thermal cycling, or salivary exposure. As a result, the durability of antifungal effects under real-world conditions remains uncertain. These limitations highlight that current models provide proof-of-concept, rather than clinically translatable evidence.

### 4.3. Single-Agent Versus Synergistic Effects

A key mechanistic observation is the distinction between intrinsic (single-agent) effects of selenium and synergistic effects resulting from functionalization. Selenium nanoparticles alone exhibit antifungal activity through mechanisms including membrane disruption, oxidative stress induction, and interference with fungal metabolism [44]. However, mechanistic studies consistently demonstrate enhanced efficacy when selenium is combined with functionalizing agents such as chitosan or phytochemicals [15,16,17,51]. These combinations improve nanoparticle stability, increase interaction with fungal cell walls, and enable modulation of virulence pathways, including those associated with hyphal transition and biofilm maturation [14,15,16,17]. Despite this, the evidence for synergy is derived almost exclusively from mechanistic studies rather than denture material experiments. Direct comparisons between single-agent and synergistic formulations on prosthetic substrates are lacking. Consequently, while synergy represents a plausible mechanism, its magnitude and clinical relevance in denture applications remain uncertain.

### 4.4. Strengths and Limitations of the Evidence

The principal strength of the available evidence lies in the consistency of antifungal effects across independent in vitro studies, despite differences in nanoparticle synthesis and experimental design. The absence of measurable adverse effects on denture material properties and the reported stability of selenium incorporation further support the feasibility of this approach [19,23,45]. However, several limitations significantly constrain interpretation. First, the evidence base is small, with only four studies directly evaluating denture materials. Second, all included studies are in vitro, with no animal models or clinical trials available. Third, there is substantial heterogeneity in nanoparticle synthesis, functionalization, and characterization, making it difficult to define optimal formulations. Finally, methodological limitations, including incomplete reporting and lack of bias-control measures, reduce reproducibility. Taken together, these factors indicate that the current evidence should be interpreted as preclinical and exploratory, rather than confirmatory.

### 4.5. Mechanistic Plausibility in the Context of Denture Biofilms

Mechanistic studies provide a coherent biological rationale for the observed antifungal effects. Selenium nanoparticles act through multiple pathways, including disruption of cell membranes, inhibition of biofilm matrix formation, and modulation of virulence-related gene expression [14,15,44]. These mechanisms are relevant to *Candida* biofilms on prosthetic surfaces, which are characterized by enhanced resistance to conventional antifungal agents and complex structural organization [1,11].

However, it is important to emphasize that these mechanistic findings were not obtained in denture-specific systems. Therefore, they should be interpreted as supporting evidence, rather than direct proof of efficacy in clinical prosthetic environments.

### 4.6. Safety and Biocompatibility

The included studies provide limited but consistent evidence of short-term cytocompatibility, particularly for functionalized selenium nanoparticles [13,14,15,16,17]. In denture material studies, no measurable selenium leaching was reported under experimental conditions, suggesting stable incorporation within the material matrix [19,52]. These findings are relevant but insufficient to establish safety. Data on long-term exposure, chronic toxicity, mucosal interactions, and systemic absorption are lacking. Evidence from non-oral models indicates that selenium toxicity is dose- and formulation-dependent, and may involve systemic effects under prolonged exposure [55]. These aspects were not addressed in the included studies and remain speculative in the context of denture applications. Therefore, current conclusions regarding safety should be limited to short-term in vitro observations, with no extrapolation to clinical use.

### 4.7. Translational Gaps and Future Directions

Several key gaps must be addressed before clinical application can be considered.

First, standardization is required across nanoparticle synthesis, functionalization, and characterization protocols to ensure reproducibility and comparability [59]. Second, clinically relevant preclinical models are needed, including polymicrobial biofilms and in vivo denture systems that replicate oral environmental conditions [57,58,75,76]. Third, long-term stability and safety must be evaluated, including aging of denture materials and assessment of selenium release kinetics. Finally, randomized clinical trials are necessary to determine clinical efficacy, safety, and patient-centered outcomes.

Without addressing these steps, translation into clinical practice remains premature.

### 4.8. Clinical Interpretation

Functionalized selenium nanoparticles and organoselenium-modified denture materials demonstrate promising antifungal and antibiofilm activity in preclinical models [13,15,44,51]. The observed effects are consistent across different materials and formulations, and are supported by plausible biological mechanisms. However, given the exclusively in vitro nature of the evidence, the heterogeneity of experimental models, and the absence of long-term safety and clinical data, no clinical recommendations can currently be made. These strategies should be regarded as experimental approaches with potential translational relevance, rather than established therapeutic or preventive interventions.

## 5. Conclusions

Biogenic selenium nanoparticles functionalized with natural polymers or phytochemicals demonstrate promising antibiofilm activity against *Candida* biofilms on denture materials in in vitro models, with generally favorable short-term biocompatibility and no consistent adverse effects on material properties. However, the current evidence base is limited to preclinical studies, is methodologically heterogeneous, and lacks long-term safety data. Only a small number of studies directly evaluated denture materials, while the majority provide indirect mechanistic support. Future research should prioritize standardized nanoparticle synthesis and characterization protocols, the development of validated in vivo denture models incorporating polymicrobial biofilms, and well-designed clinical trials to establish efficacy, safety, and translational relevance.

## Figures and Tables

**Table 1:** Search syntax used in the study.

Source	Search Syntax	N
PubMed	(“selenium nanoparticle*” OR SeNP* OR nano-selenium OR nanoselenium OR organoselenium OR “selenium nanomaterial*” OR “selenium nanostructure*” OR “Se nanoparticle*”) AND (Candida OR “Candida albicans” OR “Candida spp*” OR yeast OR fungal) AND (biofilm* OR adhesion OR colonization OR antibiofilm OR “anti-biofilm” OR antifungal OR antimicrobial) AND (denture* OR PMMA OR “polymethyl methacrylate” OR “denture base*” OR “dental resin*” OR “acrylic resin*” OR prosthodontic* OR “oral prosthe*” OR stomatitis OR oral) AND (biogenic OR biosynthes* OR “green synthes*” OR phytosynthes* OR “biological synthes*” OR “microbial synthes*” OR plant-mediated OR fungal-mediated OR bacterial-mediated OR chitosan OR polysaccharide* OR “plant extract*” OR phytochemical* OR biopolymer* OR “natural polymer*” OR alginate OR pectin OR cellulose OR polymer* OR biomaterial* OR coating OR composite)	28
Embase	(‘selenium nanoparticle*’ OR senp* OR ‘nano selenium’ OR nanoselenium OR organoselenium OR ‘selenium nanomaterial*’ OR ‘selenium nanostructure*’ OR ‘se nanoparticle*’):ti,ab,kw AND (candida OR ‘candida albicans’ OR ‘candida spp*’ OR yeast OR fungal):ti,ab,kw AND (biofilm* OR adhesion OR colonization OR antibiofilm OR ‘anti biofilm’ OR antifungal OR antimicrobial):ti,ab,kw AND (denture* OR pmma OR ‘polymethyl methacrylate’ OR ‘denture base*’ OR ‘dental resin*’ OR ‘acrylic resin*’ OR prosthodontic* OR ‘oral prosthe*’ OR stomatitis OR oral):ti,ab,kw AND (biogenic OR biosynthes* OR ‘green synthes*’ OR phytosynthes* OR ‘biological synthes*’ OR ‘microbial synthes*’ OR plant mediated OR fungal mediated OR bacterial mediated OR chitosan OR polysaccharide* OR ‘plant extract*’ OR phytochemical* OR biopolymer* OR ‘natural polymer*’ OR alginate OR pectin OR cellulose OR polymer* OR biomaterial* OR coating OR composite):ti,ab,kw	6
Scopus	TITLE-ABS-KEY((“selenium nanoparticle*” OR SeNP* OR “nano selenium” OR nanoselenium OR organoselenium OR “selenium nanomaterial*” OR “selenium nanostructure*” OR “Se nanoparticle*”)) AND TITLE-ABS-KEY((Candida OR “Candida albicans” OR “Candida spp*” OR yeast OR fungal)) AND TITLE-ABS-KEY((biofilm* OR adhesion OR colonization OR antibiofilm OR “anti biofilm” OR antifungal OR antimicrobial)) AND TITLE-ABS-KEY((denture* OR PMMA OR “polymethyl methacrylate” OR “denture base*” OR “dental resin*” OR “acrylic resin*” OR prosthodontic* OR “oral prosthe*” OR stomatitis OR oral)) AND TITLE-ABS-KEY((biogenic OR biosynthes* OR “green synthes*” OR phytosynthes* OR “biological synthes*” OR “microbial synthes*” OR “plant mediated” OR “fungal mediated” OR “bacterial mediated” OR chitosan OR polysaccharide* OR “plant extract*” OR phytochemical* OR biopolymer* OR “natural polymer*” OR alginate OR pectin OR cellulose OR polymer* OR biomaterial* OR coating OR composite))	11
Cochrane Library	(“selenium nanoparticles” OR SeNPs OR nano-selenium OR organoselenium) AND (biogenic OR biosynthesis OR “green synthesis” OR microbial OR plant-mediated OR fungal-mediated) AND (chitosan OR polysaccharide OR phytochemical OR biopolymer OR alginate OR pectin OR cellulose) AND ((Candida OR “Candida albicans”) AND (biofilm OR adhesion OR colonization OR antibiofilm)) AND (denture OR PMMA OR “polymethyl methacrylate” OR “acrylic resin” OR “denture stomatitis”))	3

**Table 2:** Summary of denture-related and supporting studies evaluating selenium-based antifungal strategies.

Author, Year	Study Category	Antifungal/Biofilm Outcomes	Material Property Effects	Stability/Leaching Results	Authors’ Main Conclusions
Gao et al., 2025 [41]	Primary Denture Study	Total eradication of mature biofilms on denture base.	Enhanced wear resistance of the material.	High retention after mechanical brushing.	Synergy of SeNPs and 3D resin is potentially translatable.
Mirani et al., 2025 [42]	Primary Denture Study	Marked reduction in *Candida* biomass and CFU counts on PMMA resin.	Improved antibiofilm performance; mechanical properties maintained.	SeNPs stably incorporated; no measurable nanoparticle release.	Biosynthesized SeNPs enhance antibiofilm functionality of PMMA for dental use.
Hamed et al., 2025 [43]	Mechanistic Support Study	Metabolic enzyme inhibition leading to potent antifungal effects.	Not assessed.	Stable Se/chitosan nano-incorporates.	Antimicrobial activity is mediated by metabolic pathways and oxidative stress.
Thombre et al., 2024 [44]	Mechanistic Support Study	Strong inhibition of hyphal transition and virulence factors.	Not assessed.	Stable phytochemical-functionalized SeNPs (Ginger extract).	Ginger-functionalized SeNPs target *Candida* virulence with low host toxicity.
AlMojel et al., 2023 [45]	Primary Denture Study	Significant inhibition of *C. albicans* biofilm growth on treated surfaces.	No adverse effects on denture surface integrity or topography.	Stable organoselenium coating; no detectable leaching.	Organoselenium sealants effectively inhibit biofilms without compromising materials.
Tritean et al., 2023 [46]	Mechanistic Support Study	Broad antimicrobial activity including *Candida* spp. in hydrogel form.	Hydrogel demonstrated favorable mucoadhesive properties.	High nanoparticle stability; no cytotoxic leaching.	Phytosynthesized SeNPs are cytocompatible and biologically active in oral environments.
Nile et al., 2023 [47]	Mechanistic Support Study	Inhibition of biofilm via nystatin-functionalized SeNPs.	Not assessed.	Surface modification identified as a key factor for stability.	Synergy between SeNPs and antifungal drugs increases treatment efficacy.
Tran et al., 2022 [48]	Primary Denture Study	Prevention of fungal adhesion on acrylic surfaces.	Improved surface hydrophilicity.	Stable covalent bonding to PMMA.	Organoselenium coatings prevent initial colonization.
Hosseini Bafghi, 2022 [49]	Mechanistic Support Study	Effective against drug-resistant clinical isolates of *Candida*.	Not assessed.	High zeta potential indicating excellent colloidal stability.	Green SeNPs are a viable alternative to conventional antifungals.
Song et al., 2021 [50]	Mechanistic Support Study	Structural damage to fungal cell walls and ROS generation.	Improved physicochemical properties of polymer films.	High stability in polysaccharide-based systems.	Natural polymers (gum Arabic, carrageenan) are optimal for SeNP functionalization.
Lara et al., 2018 [51]	Mechanistic Support Study	Synergistic activity against mature *Candida* biofilms; cell wall distortion.	Not assessed (nanoparticle focus).	Chitosan-stabilized SeNPs showed high colloidal stability.	Chitosan-functionalized SeNPs enhance efficacy via membrane-disrupting synergy.

**Table 3:** Risk of Bias assessment in the included studies.

Study	Biogenic Synthesis Characterization	Physicochemical Characterization	Substrate Standardization	Biofilm Model Description	Appropriate Comparators	Validated Outcome Measures	Bias-Control Measures	Statistical Analysis	Transparency COI	Total Score	Overall Risk of Bias
Gao et al., 2025 [41]	1	1	1	1	1	1	0	1	1	8/9	Low
Mirani et al., 2025 [42]	1	1	1	1	1	1	0	1	1	8/9	Low
Hamed et al., 2025 [43]	1	1	0	1	1	1	0	0	1	6/9	Moderate
Thombre et al., 2024 [44]	1	1	0	1	1	1	0	0	1	6/9	Moderate
AlMojel et al., 2023 [45]	1	1	0	1	1	1	0	1	1	7/9	Low
Tritean et al., 2023 [46]	1	1	0	1	1	1	0	1	1	7/9	Moderate
Nile et al., 2023 [47]	1	1	0	1	1	1	0	0	1	6/9	Moderate
Tran et al., 2022 [48]	1	1	1	1	1	1	0	1	1	8/9	Low
Hosseini Bafghi, 2022 [49]	1	0	0	1	1	1	0	0	1	5/9	Moderate
Song et al., 2021 [50]	1	1	0	1	1	1	0	1	0	6/9	Moderate
Lara et al., 2018 [51]	1	1	0	1	1	1	0	0	1	6/9	Moderate

## Data Availability

The original contributions presented in the study are included in the article, further inquiries can be directed to the corresponding authors.

## Supplementary Materials

The following supporting information can be downloaded at: https://www.mdpi.com/article/10.3390/jfb17050216/s1, File S1: PRISMA Flowchart. Reference [77] is cited in the Supplementary Materials.
